# Effect of Extender and Equilibration Time on Post Thaw Motility and Chromatin Structure of Buffalo Bull (Bubalus Bubalis) Spermatozoa

**Published:** 2014-10-04

**Authors:** Abdolhossain Shahverdi, Abdolreza Rastegarnia, Tohid Rezaei Topraggaleh

**Affiliations:** 1 Department of Embryology at Reproductive Biomedicine Research Center, Royan Institute for Reproductive Biomedicine, ACECR, Tehran, Iran; 2 Department of Clinical Science, Faculty of Veterinary Medicine, Urmia Branch, Islamic Azad University, Urmia, Iran; 3 Faculty of Veterinary Medicine, Urmia Branch, Islamic Azad University, Urmia, Iran

**Keywords:** Buffalo, Sperm, Cryopreservation, Extender, Chromatin

## Abstract

**Objective:**

The aim of the present study was to investigate the effects of four equilibration times (2, 4, 8 and 16 hours) and two extenders (tris or Bioxcell®) on cryopreservation of buffalo semen.

**Materials and Methods:**

In this experimental study, split pooled ejaculates (n=4), possessing more than 70% visual sperm motility were divided in two aliquots and diluted in Bioxcell® and tris-citric egg yolk (TCE) extenders. Semen was cooled to 4°C within 2 hours, equilibrated at 4°C for 2, 4, 8 and 16 hours, then transferred into 0.5 ml French straws, and frozen in a programmable cell freezer before being plunged into liquid nitrogen. Postthaw motility characteristics, plasma membrane integrity, acrosome morphology and DNA integrity of the buffalo sperm were studied after thawing.

**Results:**

There were significant interactions between equilibration times and extenders for sperm motility and membrane integrity. Post thaw sperm motility (PMOT), progressive motile spermatozoa (PROG), plasma membrane integrity (PMI) and normal apical ridge (NAR) measures were lower for sperm equilibrated for 2 hours in both TCE and Bioxcell® extender compared to others equilibration times. PMOT, PMI and NAR for sperm equilibrated for 4, 8 and 16 hours showed no significant differences in either extender, although PROG measures were superior in Bioxcell®compared to TCE at all equilibration times (p<0.05). Kinematic parameters such as average path velocity, curvilinear velocity and linearity in the Bioxcell®extender were superior to those in the TCE extender studied. In contrast to motility and viability, the DNA integrity of post thaw spermatozoa remained unaffected by different equilibration times.

**Conclusion:**

Equilibration time is necessary for preservation of the motility and integrity of buffalo sperm membranes. Equilibration times of over than 2 hours resulted in the greatest preservation of total semen parameters during cryopreservation. There were no significant interactions between equilibration times over 4 hours and type of extender which lead to greater post thaw sperm survival.

## Introduction

The artiﬁcial insemination (AI) industry has been always interested in improving the quality of the frozen semen marketed (1). Protocols for freezing bull semen usually include slow cooling to 4-5˚C, followed by a variable interval of equilibration (from 30 minutes to 24 hours) at this low temperature before freezing (2). The traditional definition of equilibration is the total time during which, spermatozoa remain in contact with glycerol before freezing. At this stage, glycerol penetrates into the sperm cell to establish a balanced intracellular and extracellular concentration. It should not be overlooked that the equilibration process applies not only to glycerol, but also to the other osmotically active extender components. Therefore, the equilibration process can interacts with the type of extender (buffer and cryoprotectant) used and could easily interact with other cryogenic procedures (1, 3).

Freezing and thawing followed by equilibration causes maximum damage to the motility apparatus, plasma membrane, and the acrosomal cap of buffalo spermatozoa (4). Further studies are in progress to minimize these damages by altering the equilibration time and the rate of freezing for buffalo spermatozoa (5-7). Glycerol and several low density lipoproteins from egg yolk are routinely used for the cryopreservation of buffalo semen (8, 9). Most cryopreservation protocols for buffalo sperm use an equilibration period of 4 hours, thus, the semen has to be frozen on the same day of collection (7, 9, 10).

It is noteworthy that the results of several fertility trials designed to determine the optimal equilibration period for bull semen established the beneﬁcial effect of a period of several hours (4-17) at 5˚C before freezing to obtain maximal fertility (11). However, several other studies have indicated that an equilibration period of 18 hours or overnight before freezing resulted in increased and semen quality and fertility in bulls (7, 11).

Such prolonged periods of equilibration are very convenient for the working schedule in AI centers. A high number of bull ejaculations are collected each day, and it is more practical to freeze all the semen collected on the morning of the next day. Most of the studies that have included prolonged equilibration periods were done using egg yolk or milk-based extenders for bull and buffalo semen (8, 9, 12, 13).

There is disagreement regarding the necessity and duration of equilibration on semen effect on cryopreservation and its sperm viability. In addition, there is a desire to shorten or eliminate this step, hastening cryopreservation without compromising post-thaw sperm quality (13-15).

Glycerol and egg yolk are the most commonly used cryoprotectants, but in recent years there has been a trend against the use of egg yolk in cryoprotective media, due to sanitary risks. As a consequence, a well-deﬁned and pathogen-free, non-animal origin substitute for yolk was needed and soybean lecithinbased extenders were developed. To date, however, little is known about their interactions with equilibration time for buffalo semen (16-18).

A few investigators have used subjective assessments of semen in an attempt to establish the optimal duration of equilibration for soybean lecithin- based extenders. However, these produced conﬂicting reports regarding survival and fertility of frozen-thawed buffalo semen, particularly when working with these extenders, semen packaging, and rates of cooling and freezing (5, 19, 20). Earlier reports have shown that the prolonged cooling during equilibration (4 hours at 4˚C) seems to change the permeability of buffalo sperm by decreasing the plasma membrane integrity after equilibration, freezing and thawing. In addition, premature capacitation-like changes might occur, similar to bull spermatozoa (19, 20).

To our knowledge, there are no reports on optimal the freezing protocols for using soybean lecithin extenders in combination with a prolonged equilibration period for buffalo semen. The objectives of the present research were to investigate further the effects of short and long equilibration times and the type of extenders, and interactions, between time and extender on the cryopreservation of buffalo semen, based on motility, integrity of plasma and acrosomal membranes, using objective and precise methods, i.e. Computer-Assisted Semen Analysis (CASA) and flow cytometry in buffalo spermatozoa.

## Materials and Methods

### Preparation of extenders

The experiment was conducted at the Buffalo Breeding and Extension Training Center, Urmia, West Azerbaijan, Iran (Latitude: 38́ 23″ N, Longitude: 47́ 40″ E, Altitude: 1568.5 M) during October and December 2010. Tris-citric egg yolk extender was prepared by using 3.0 g tris-(hydroxymethyl-aminomethane) and 1.56 g citric acid, fructose 0.2% w/v, glycerol 7.0 ml (Merck, Germany), and egg yolk 20% in 74 ml distilled water. All chemicals used in this study were obtained from Sigma-Aldrich (St. Louis, MO, USA) unless otherwise indicated. Antibiotics namely benzyl penicillin (1000 IU/ml, Pharmacia & Upjohn, Belgium) streptomycin sulphate (1000 μg/ml, Pharmacia & Upjohn, Belgium) were added to tris-citric egg yolk extender. Bioxcell® as soybean lecithin extenders was prepared according to manufacturer’s instructions (IMV, France).

### Semen collection and freezing

Semen was collected (two consecutive ejaculates/ bull/week) using artificial vagina (IMV, France), (at 42℃) from four adult buffalo bulls (Bubalus bubalis) of known fertility and similar age (4-5 years) for a period than six weeks (replicate). Ejaculated semen from each bull was immediately transferred to the laboratory. Sperm progressive motility was determined microscopically (×400, Olympus BX20, Tokyo, Japan) and sperm concentration was determined using a digital photometer (IMV, France). At least, one ejaculate from each bull at each replicate always passed the criteria (motility >70%). To eliminate individual differences, semen samples from the four bulls were pooled. Each pooled sample was split into two aliquots and diluted with extender Bioxcell® or Tris-citric egg yolk (TRIS) at 37℃, added in single step for a final concentration of 40×10^6^ sperm/mL. The semen extension was performed immediately after the sperm motility and concentration evaluations. After dilution, semen was maintained in a water bath for 10 minutes at 35℃ for stabilization; thereafter, it was cooled from 37 to 25℃ in approximately 1 hour at room temperature (22-25℃). Straws designated for the same duration of equilibration time were transferred to the same freezing procedure. Freezing rates (20℃/min-from 5 to, 120℃-duration: 10 minutes), varying only for the equilibration time at 5℃: 2 hours (T1), 4 hours (T2), 8 hours (T3) and 16 hours (T4), for a total of four treatments. French straws (IMV, France) with suction pump at 4℃ in a cold cabinet unit (IMV, France) and placed in liquid nitrogen vapors, 5 cm above the level of liquid nitrogen. Straws were then plunged and stored under liquid nitrogen (196℃). After 72 hours, four frozen straws from each group were thawed individually at 37℃ for 30 seconds in a water bath for evaluation.

### Semen evaluation

Semen analysis was conducted in the Department of Embryology and Reproductive Medicine Research Center of the Royan Institute.

### Motility

An aliquot of semen (5 µL) was placed on a prewarmed (37℃) Makler chamber (depth 10 µm) and analyzed for sperm motion characteristics using a computer-assisted sperm analyzer (Sperm Class Analyzer, Microptic, Barcelona, Spain). The CASA-derived motility characteristics were analyzed immediately after thawing and four hours of incubation at 37℃. Four microscopic fields were analyzed in each sample using a phase-contrast microscope (Nikon, Tokyo, Japan) supplied with a prewarmed stage at 37℃ and at ×100 magnification. A total of four microscopic fields with 400 spermatozoa were analyzed. Objects incorrectly identified as spermatozoa were minimized on the monitor by using the playback function. Total motility was defined as the percentage of spermatozoa with mean velocity (VAP) above 10 µm/s. The CASA derived motility characteristics studied were percentages of motility and progressive motility, straight-line velocity (VSL, µm/s, the straight-line distance from beginning to end of track divided by time taken), average path velocity (VAP, µm/s, the spatial averaged path that eliminated the wobble of the sperm head), curvilinear velocity (VCL, µm/s, total distance traveled by a sperm during the acquisition divided by the time taken), lateral head displacement (LHD, µm, deviation of the sperm head from the average path), linearity (LIN, %, VSL/VCL ×100), straightness(STR, %, VSL/VAP ×100), lateral amplitude (ALH, µm/s, maximum amplitude of lateral head displacement) and beat central frequency (BCF, Hz, beat frequency of centroids crossing the average trajectory) (21).

Sperm plasma membrane integrity was determined using a hypo-osmotic swelling (HOS) assay. HOS solution consisted of 0.73 g sodium citrate and 1.35 g fructose dissolved in 100 ml distilled water (osmotic pressure:-190 mOsmol/ Kg). To assess the sperm tail plasma membrane integrity, semen (50 μl) was mixed with HOS solution (500 μl) and incubated for 30 minutes at 37˚C before examination with a phase contrast microscope (×400, Olympus BX20, Tokyo, Japan). Two hundred spermatozoa were assessed for their swelling ability in HOS. The swollen spermatozoa characterized by coiling of the tail were considered to have an intact plasma membrane (22).

### Normal acrosomes

To assess sperm acrosomal integrity, 100 μl of semen sample was fixed in 500 μl of 1% formal citrate (2.9 g tri-sodium citrate dihydrate, 1 ml of 37% solution of formaldehyde, dissolved in 100 ml of distilled water); one hundred spermatozoa were examined with a phase contrast microscope (×1000, Olympus BX20, Tokyo, Japan) under oil immersion. A normal acrosome was characterized by normal apical ridge (8).

### Assessment of DNA integrity

Chromatin stability was assessed by using the sperm chromatin structure assay (SCSA) technique. This technique is based on the susceptibility of the sperm DNA to acid induced denaturation in situ and metachromatic staining by acridine orange (AO). AO shifts from green (dsDNA) to red (ssDNA) fluorescence depending on the degree of DNA denaturation. After thawing at 37˚C for 30 seconds, samples were diluted with Tris-Null-EDTA (TNE) buffer (0.01 m Tris-HCl, 0.15 m NaCl, 1 mm EDTA, pH=7.4) in cryotubes, at a final sperm concentration of 20×10^6^ cells/ml. A 100 μl aliquot of this suspension was mixed with 200 μl of a detergent/ acid solution (0.1% v/v Triton X-100 in 0.08 MHCl, 0.15 MNaCl). After 30 seconds, 0.6 ml of an acridine orange solution (6 μg/ml of acridine orange in 0.15 M NaCl, 1mM EDTA, 0.2 M Na_2_HPO_4_, 0.1 M citric acid, pH=6.0) was added to the sample and the cells were subjected immediately to flow cytometry after 30 minutes incubation at room temperature (23, 24).

### Flow cytometer analysis

Flowcytometric analysis was performed using FACS Calibur (BD Immunocytometry Systems, San Jose, Jose, CA, USA) with an air-cooled argon laser operated at 488 nm excitation and 15 mW. For the acridine orange assay, the green fluorescence (intact DNA) detected by the FL-1 detector (515/45-nm band-pass filter) was compared with red fluorescence (single-stranded DNA) detected by the FL-3 detector (640 nm long-pass filter) after gating out non-sperm and aggregated events. Ten thousand sperm cells were acquired and analyzed in each sample at the rate of 1000 events per second and analyzed further with cytologic software (Cyflogic version 1.2.1).

### Statistical analysis

A completely randomized block design in a 2×4 factorial arrangement (2 extenders ×4 equilibration times), with 12 replications per experimental unit was used. For assessment of DNA fragmentation, each treatment consisted of at least six replicates. Results are presented as mean ± standard deviation. Effects of extender and equilibration time were evaluated by ANOVA, with means compared by Duncan’s test at a 5% level. All the statistical analyses were performed using the SAS software (version 9.0, SAS Institute Inc., USA), and differences were considered significant at p<0.05 level.

## Results

### Motility

The type of extender did not have a significant effect on overall post thaw sperm motility as determined subjectively (Table 1). The post thaw sperm motility and the percentage of progressive motile spermatozoa after 2 hours equilibration in both TCE and Bioxcell® extenders were both lower than for the other equilibration times. However, these indices were superior for the soybean lecithin-based extenders compared to the TCE extenders at the two- hour equilibration time. On the other hand, post thaw sperm motility for equilibration times of 4, 8 and 16 hours did not show significant differences in either extender. However, the percentage of progressive motile spermatozoa was higher in the soybean lecithin-based extender than in the TCE extender at all times (p<0.05). Kinematic parameters such as VSL, VCL, VAP and LIN were superior in the soybean lecithin-based extender compared to the TCE extenders studied. However, other, others kinematic parameters such as ALH and BCF were superior in TCE extender compared to the soybean lecithin-based extender (Table 2).

**Table 1:** Effect of different extenders and equilibration time on motility and kinematics sperm parameters for the buffalo semen samples thawed at 37˚C


Variable	Extender	Equilibration time			
		T2	T4	T8	T16

**Total motility**	TRIS	15.3 ±5.81ᵃ^,^ᶠ	60.1 ±6.98 ᵇ	56.2 ±8.61ᵇ	54.1 ±10.3ᵇ
	BIO	30.4 ±9.38 ᶜ^,^ᵉ	56.7 ±7.23 ᵇ	51.9 ±5.22ᵇ	53.1 ±7.13ᵇ
** PM (%)**	TRIS	4.98 ±3.39 ᵃ^,^^f^	32.4 ± 6.77 ᵇ	31.4 ±3.79 ᵇ	28.3 ±5.49 ᵇ
	BIO	16.9 ±6.95 ᶜ^,^ᵉ	40.1 ±8.67 ᵃ	36.2 ±5.31 ᵃ	35.5 ±9.01ᵃ


**Table 2:** Effect of different extenders and equilibration time on kinematics sperm parameters for the buffalo semen samples thawed at 37˚C


variable	extender	equilibration time			
		T2	T4	T8	T16

**VSL (m/s)**	TRIS	14.7 ±5.11ᵃ^,^ᶠ	24.5 ±7.26 ᵃ	29.3 ±7.59ᵃ	20.7 ±3.48ᵃ
	BIO	60.5 ±12.3 ᵇ^,^ᵉ	66.1 ±12.2ᵇ	60.2 ±14.1ᵇ	59.8 ±19.5ᵇ
**VCL (m/s)**	TRIS	38.5 ±9.83ᵃ^,^ᶠ	45.8 ±5.67ᵃ	48.7 ±3.11ᵃ	46.1 ±6.35ᵃ
	BIO	81.7 ±14.1ᵇ^,^ᵉ	85.7 ±16.6ᵇ	78.5 ±18.1ᵇ	77.8 ±24.1ᵇ
**VAP (m/s)**	TRIS	20.7 ±6.16 ᵃ^,^ᶠ	36.0 ±17.1ᵃ	41.7 ±16.8ᵃ	27.3 ±4.37ᵃ
	BIO	72.7 ±14.3 ᵇ^,^ᵉ	77.5 ±16.1ᵇ	70.3 ±17.7ᵇ	69.6 ±23.1ᵇ
**LIN**	TRIS	36.6 ±7.71ᵃ^,^ᶠ	47.9 ±5.45ᵃ	52.2 ±4.81ᵃ	44.5 ±3.08ᵃ
	BIO	73.7 ±4.94 ᵇ^,^ ᵉ	78.5 ±4.86ᵇ	76.7 ±1.97ᵇ	76.4 ±3.75ᵇ
**STR (%)**	TRIS	67.9 ±9.81ᵃ	73.5 ±6.14ᵃ	76.7 ±4.73ᵃ	75.2 ±2.69ᵃ
	BIO	83.4 ±4.11ᵇ	85.7 ±3.74ᵇ	85.9 ±2.11ᵇ	86.1 ±2.43ᵇ
**ALH**	TRIS	2.30 ±0.87ᵃ	2.22 ±0.29ᵃ	2.43 ±0.21ᵃ	2.65 ±0.38ᵃ
	BIO	2.38 ±0.23ᵃ	2.15 ±0.32ᵃ	2.14 ±0.17ᵃ	2.12 ±0.36ᵃ
**BCF (Hz)**	TRIS	8.15 ±3.89 ᵃ^,^ᶠ	9.65 ±1.02ᵃ	9.71 ±0.38ᵃ	9.60 ±0.31ᵃ
	BIO	7.86 ±0.62 ᵇ^,^ ᵉ	7.66 ±0.63ᵇ	7.38 ±0.66ᵇ	7.67 ±0.81ᵇ


### Comparison of post-thaw sperm viability, plasma membrane integrity and acrosomal ridge

The data on plasma membrane integrity and acrosomal ridge of buffalo bull spermatozoa are given in table 3. After thawing, the proportions of sperm that retained plasma membrane integrity and a normal apical ridge were lower for the twohour equilibration group in both TCE and Bioxcell extenders than for the rest of the equilibration times. However, the percentages of sperm that retained plasma membrane integrity (33.5 ± 6.55 vs. 46.7 ± 3.31) and normal acrosomal ridges (35.2 ± 4.78 vs. 58.7 ± 8.46) were superior in the soybean lecithin-based extenders than in the TCE extender for this equilibration time, respectively (p<0.05). On the other hand, the data on post thaw plasma membrane integrity and acrosomal ridge of buffalo bull spermatozoa for equilibration times of 4, 8 and 16 hours did not show significant differences in either the TCE and Bioxcell extenders (p>0.05, Table 3). The type of extender as well as the interaction between extender and equilibration time had no significant effects.

**Table 3:** Effect of different extenders and equilibration time on viability, plasma membrane integrity (PMI) , normal apical ridge (NAR) and DNA damage for the Buffalo bull semen samples thawed at 37˚C


variable	extender	equilibration time			
		T2	T4	T8	T16

**PMI (%)**	TRIS	33.5 ±6.55ᵇ^,^ᵉ	62.7 ±6.85ᵃ	63.5 ±6.55ᵃ	57.8 ±4.96ᵃ
	BIO	46.7 ±3.31ᶜ^,^ᶠ	68.7 ±5.47ᵃ	62.2 ±6.18ᵃ	59.2 ±1.75ᵃ
**NAR (%)**	TRIS	35.2 ±4.78ᵇ^,^ᵉ	68.0 ±4.96ᵃ	69.5 ±5.91ᵃ	62.5 ±3.31ᵃ
	BIO	58.7 ±8.46ᶜ^,^ᶠ	67.0 ±5.47ᵃ	62.7 ±8.54ᵃ	64.5 ±4.79ᵃ
**DNA damage **	TRIS	6.88 ±0.51ᵃ	4.51 ±0.72ᵃ	5.91 ±0.82ᵃ	5.71 ±0.93ᵃ
	BIO	6.74 ±0.31ᵃ	4.72 ±0.31ᵃ	5.97 ±0.90ᵃ	5.61 ±0.98ᵃ


### Comparison of post-thaw sperm DNA integrity

**Fig 1:**
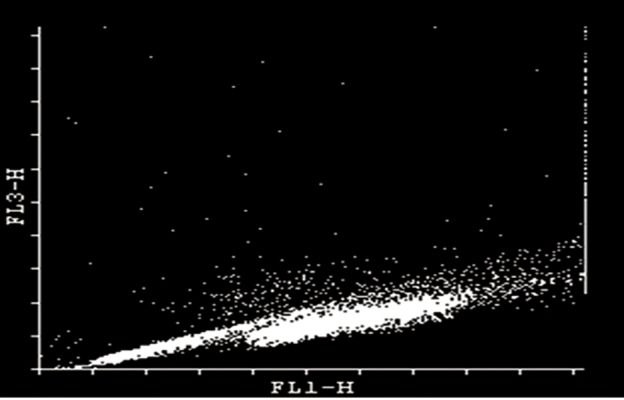
Example of SCSA cytogram of individul buffalo post thaw sperm cells. Each cell’s position is based on the amount of nativ DNA satiability (green fluorescence; FL 1) vs. fragmented DNA (red fluorescence; FL 3).

Chromatin damage of each sperm was quantified by red fluorescence. Each semen sample contained a percentage of mature cells with non-detectable (main population of spermatozoa in semen) and a percentage with detectable (mature spermatozoa with increased chromatin damage) damage (Fig 1). The equilibration time had not significant effect of the percentage on spermatozoa with damaged DNA as determined subjectively (Table 3, p>0.05). The overall mean DNA damage in the two- hour equilibration group was (6.88 ± 0.51 and 6.74 ± 0.31) for the Bioxcell and TEC extenders, respectively (p>0.05). In contrast with motility and viability, the DNA integrity of post thaw spermatozoa remained unaffected across the different equilibration times. It was (4.51 ± 0.72, 5.91 ± 0.82, 5.71 ± 0.93%) and (4.72 ± 0.31, 5.97 ± 0.90, 5.61 ± 0.98%) for the equilibration times 4, 8 and 16 hours for the Bioxcell and TEC extenders, respectively (p>0.05, Table 3).

## Discussion

The present study showed that there were significant interactions between equilibration times and extenders for sperm motility and membrane integrity. However, the equilibration time did not have a significant effect on the percentage of spermatozoa with damaged DNA determined subjectively. In a similar study of the cryopreservation of bull semen, Leite et al (2) reported that the use of zero equilibration time, in comparison to 2 and 4 hours, gave the lowest values for total and progressive sperm motility, and percentage of sperm with intact plasma and acrosomal membranes, with no significant differences between tris and Bioxcell ® extenders.

Most cryopreservation protocols for buffalo sperm suggest an equilibration period of 4 hours, thus, the semen has to be frozen on the same day of collection (7, 10, 20, 23, 25-27). Tuli et al. (27) reported that buffalo sperm survivability in Tris egg-yolk glycerol extender was found to be better at all the stages of deep-freezing using 4 hours compared to 0 and 2 hours equilibration time. However, Dhami et al. (13) reported that 2 hours of equilibration at 5℃ compared with 0 hour improved the post-thaw recovery, incubation survival and fertility rates of buffalo frozen semen. In other studies, Sukhato et al. (28) used an equilibration period time of less than 1h, and Adeel et al. (29) use of an equilibration period of 6 hours, for cryopreservation buffalo semen. It is pertinent to mention that, mammalian spermatozoa can be stored at 5℃ for at least 24 hours without signi.cant reduction in motility and fertility (9, 30). Contrary to these results, in the cryopreservation of bull semen some workers have found higher conception rates for bull semen frozen following 12 to 18 hours of equilibration compared with 4 to 6 hours (12, 31, 32). Some workers have suggested shortening equilibration time to less than 2 hours for freeze bull and buffalo semen without affecting on freezability or fertility (14, 28). Muino et al. (1) compared several soybean lecithin extenders for cryopreservation of bull semen using an equilibration time of over 18 hours and reported that, when holding the semen overnight before freezing, after the use of Biladyl (as commercial egg yolk extender) results in higher sperm survival and longevity than the use of soybean lecithin extenders such as Andromed or Biociphos. Earlier reports have shown that, the recommendation made for the cryopreservation of semen of more than 7 hours equilibration to be too high (10, 33). Herold et al. (34) 2006 reported that post-thaw quality of buffalo epididymal sperm was not affected by varying the equilibration time (range, 2-9 hours) after use of soybean lecithin extenders. Foot and Kaproth (12) compared the fertility obtained when using whole milk-glycerol semen extender with and without fructose after 4 versus 18 hours of equilibration at 5℃. As there was no difference in fertility, it would appear that programs to freeze sperm in whole milk extenders the same day of collection or the day after semen collection should yield equivalent results.

Anzar et al. (34) showed that post thaw sperm quality in bull semen was greater after use of overnight equilibration as compared to 4 hours, and reported that overnight shipping of semen was found advantageous for bull semen cryopreservation. Semen packaging in 0.25 ml straws yielded better post-thaw quality than 0.5 ml straws.

Although equilibration time signi.cantly affected total and progressive motility in the present study, there was no signi.cant effect on other characteristics of sperm movement (i.e. VAP, SL, VCL, ALH, BCF, and LIN). It was noteworthy that extender had a signi.cant effect on VSL, ALH, BCF, LIN and STR; except for ALH, all these variables were greater in semen frozen with Bioxcell®. These results are in close agreement with Leite et al. (2) who observed 0 hour equilibration had the lowest values for total and progressive motilities and percentage of sperm with intact plasma and acrosomal membranes, with no signi.cant differences in kinematic parameters between extenders. However, these .ndings could have been due to differences in extender density, viscosity or even the presence of large particles, as previously suggested for bull semen (21, 34). Rasul et al. (5) reported that during prolonged equilibration, sensitive sperm undergo membrane and axonemal changes that lose their ability to move in a straight line, which results in a decrease in some kinematic parameters such as linearity, and straightness; and undergo death during freezing and thawing processes. In this study, semen cryopreserved with tris had greater BCF values, suggesting that this extender was more effective at preserving .agellar structures, or that compounds present in this extender stimulated ATP production and consequently beat frequency, as previously suggested by Celeghini et al. (35).

The present study showed that for preservation of plasma and acrosome integrity, there was a signi.cant difference between extenders only at two- hour equilibration (Bioxcell® was better), probably due to the great variation in density in semen diluted in tris extenders. There was a bene.cial effect of equilibration time for post-thaw integrity of cell membranes, and its lack was detrimental, because 2 hours had the least percentage of cells with both membranes intact and the greatest damaged plasma membrane. There were no signi.cant differences between 4, 8 and 16 hours for motility and plasma membrane integrity. The lower percentage of damaged plasma membrane cells for 8 and 16 hours indicated that a longer equilibration time was more effective at preserving the plasma membrane, independent of extender.

One of the objectives of this study was to use the Sperm Chromatin Structure Assay to determine the level and variability of damage to sperm DNA integrity in different extenders and incubation for 2,4, 8 and 16 hours. Neither of the extenders nor the equilibration time evaluated in the present study was found to have any effect on sperm chromatin structure as no significant differences were found between the Bioxcell® and and TEC extenders in terms of percentage of denatured chromatin (p>0.05).

Few studies have reported on the use of the SCSA in buffalo percentages of post thaw spermatozoa with DNA damage in our study were relatively low (<10), Similar results were found by Kadirvel et al. (23) who reported an overall mean DNA damage of 10.4% (range 4.8- 17.6) for buffalo frozen-thawed sperm. Minervini et al. (36) reported that SCSA differed signi.cantly between the buffalo bulls, however, their data showed high stability within each buffalo and DNA fragmentation indexes (DFI) were 11.2 ± 8.6. Koonjaenak et al. (37) reported that frozen–thawed swamp buffalo sperm chromatin integrity is not seriously damaged by cryopreservation or affected by the seasonal variations and the overall mean DNA fragmentation index (DFI) (±SD) was 1.84 ± 1.68%, after thawing. In other study prolonged resistance to sperm DNA fragmentation predicted better retention of fertility, but no study on the correlation between DFI parameters and fertility is reported in buffalo (23, 35).

## Conclusion

Equilibration during cryopreservation was essential for maintaining motility and integrity of sperm membranes. Equilibration times over than 2 hours resulted in the greatest preservation of total and progressive motility, as well as the integrity of plasma and acrosomal membranes during cryopreservation. As, differences regarding post thaw motility and chromatin structure for equilibration times of 4, 8 and 16 hours were non-significant. The use of 4 hours equilibration time can safe be by recommended along with soybean lecithin extender for cryopreservation of buffalo spermatozoa.
